# Methyl 3,4-dihydroxybenzoate inhibits RANKL-induced osteoclastogenesis via Nrf2 signaling *in vitro* and suppresses LPS-induced osteolysis and ovariectomy-induced osteoporosis *in vivo*: Methyl 3,4-dihydroxybenzoate inhibits osteoclastogenesis

**DOI:** 10.3724/abbs.2022087

**Published:** 2022-08-01

**Authors:** Zhaobo Huang, Zenghui Jiang, Zeyu Zheng, Xuyang Zhang, Xiaoan Wei, Jian Chen, Fengdong Zhao

**Affiliations:** 1 Department of Orthopaedics Sir Run Run Shaw Hospital Zhejiang University School of Medicine Hangzhou 310016 China; 2 Key Laboratory of Musculoskeletal System Degeneration and Regeneration Translational Research of Zhejiang Province Hangzhou 310016 China; 3 Department of Orthopaedics Xia Sha Hospital of Hangzhou City Hangzhou 310018 China; 4 Department of Orthopaedic Surgery Zhejiang Hospital Hangzhou 310030 China

**Keywords:** methyl 3,4-dihydroxybenzoate, osteoclastogenesis, Nrf2, osteoclast, osteoporosis

## Abstract

Osteoporosis deteriorates bone mass and biomechanical strength and is life-threatening to the elderly. In this study, we show that methyl 3,4-dihydroxybenzoate (MDHB), an antioxidant small-molecule compound extracted from natural plants, inhibits receptor activator of nuclear factor-κB (NF-κB) ligand (RANKL)-induced osteoclastogenesis *in vitro*. Furthermore, MDHB attenuates the activation of mitogen-activated protein kinase (MAPK) and NF-κB pathways by reducing the levels of reactive oxygen species (ROS), which leads to downregulated protein expression of c-Fos and nuclear factor of activated T cells c1 (NFATc1). We also confirm that MDHB upregulates the protein expression of nuclear factor-erythroid 2-related factor 2 (Nrf2), an important transcription factor involved in ROS regulation, by inhibiting the ubiquitination-mediated proteasomal degradation of Nrf2. Next, animal experiments show that MDHB has an effective therapeutic effect on lipopolysaccharide (LPS)- and ovariectomized (OVX)-induced bone loss in mice. Our study demonstrates that MDHB can upregulate Nrf2 and suppress excessive osteoclast activity in mice to treat osteoporosis.

## Introduction

Osteoporosis is a condition characterized by low bone mineral density (BMD) and microarchitectural deterioration of bone tissue, resulting in compromised bone strength and increased fracture risk. Osteoporosis and osteoporotic fractures have become global health issues of major concern with an increase in the aging population [1]. The prevalence of osteoporosis in China is estimated to be 29.13% in women aged 50 years and older [2]. One in three women and one in five men over the age of 50 years will experience osteoporotic fractures, which are the most serious complications of this condition [3]. Due to its dangers to public health, there is still an emerging need to develop treatments for osteoporosis.

Under the action of receptor activator of receptor activator of nuclear factor-κB (NF-κB) ligand (RANKL) and macrophage colony-stimulating factor, bone marrow monocytes/macrophages (BMMs) fuse and differentiate into multinucleated osteoclasts. Mature osteoclasts can attach to bone and exert bone resorption functions. Estrogen has antagonistic effects on osteoclasts and maintains the balance of bone metabolism under physiological conditions. In postmenopausal women, overactive osteoclasts due to lack of estrogen cause excessive bone loss in the body, leading to osteoporosis [ 4, 5] . In recent years, many studies have revealed that reactive oxygen species (ROS) are involved in the progression of osteoclast activation and that the antioxidant capacity of mesenchymal stem cells (MSCs) could help bone regeneration [ 6– 11] . Increased ROS production during RANKL-induced osteoclastogenesis can promote the activation of mitogen-activated protein kinase (MAPK) and NF-κB pathways to accelerate osteoclast differentiation [ 12, 13] . Many enzymes, such as Nox1 and HO-1, regulate osteoclast differentiation and function through their involvement in ROS production or elimination [ 12, 14] .

Nuclear factor-erythroid 2 related factor 2 (Nrf2) is a redox- related transcription factor. After translocating to the nucleus, Nrf2 participates in the gene expression of many antioxidant enzymes, resulting in the elimination of ROS [ 15– 17] . It has been reported that Nrf2 deficiency in bone marrow monocytes (BMMs) results in higher ROS levels and more osteoclasts upon RANKL stimulation [18]. Furthermore, some small molecules targeting Nrf2 have been shown to inhibit osteoclast differentiation [ 19– 22] . Due to the key roles of ROS and Nrf2 in osteoclastogenesis, it is worthwhile to identify more effective and safe drugs targeting Nrf2 to treat osteoporosis.

Methyl 3,4-dihydroxybenzoate (MDHB) is a small-molecule compound extracted from East Asian Tang Materia, Malan, and other natural plants [23]. Several studies have demonstrated that MDHB has neurotrophic, antiapoptotic, and antioxidative effects [ 24– 27] . Given the biological effects of MDHB, it may have therapeutic effects on osteoporosis related to osteoclasts. However, there are no relevant studies reporting the effect of MDHB on osteoclast activation and postmenopausal osteoporosis, which is worth exploring.

In this study, we addressed the possible role of MDHB in inhibiting osteoclastogenesis and found that MDHB suppresses osteoclast activation and function by downregulating ROS via Nrf2. Furthermore, MDHB was shown to treat osteoporosis by ovariectomy (OVX) *in vivo*. Thus far, our study is the first to clarify the negative regulatory effect of MDHB on osteoclast differentiation.

## Materials and Methods

### Reagents

MDHB was obtained from Selleck (Shanghai, China), dissolved in DMSO and stored at –20°C. Eagle’s minimal essential medium with alpha modification (α-MEM) and penicillin/streptomycin were obtained from Gibco (Carlsbad, USA). Recombinant soluble mouse RANKL and M-CSF were obtained from R&D (Minneapolis, USA). The ROS Production Detection kit was obtained from Beyotime (Shanghai, China). Antibodies against β-actin, p38, JNK, ERK, IκBα, p65, and phosphorylated ERK, JNK, p38, IκBα, p65 were obtained from Cell Signaling Technology (Boston, USA). Antibodies against CTSK, TRAP, c-Fos, Nfatc1, Nrf2, HO-1, GCLC, Nqo-1, ubiquitination (Ub) and GAPDH were obtained from Abcam (Cambridge, UK). Tartrate-resistant acid phosphatase (TRAP) staining kit was purchased from Sigma-Aldrich (St Louis, USA).

### Ethics statement

All animal experiments were overseen and approved by the Animal Ethics Committee of Sir Run Run Shaw Hospital affiliated to Zhejiang University in accordance with the principles and procedures of the National Institutes of Health (NIH) Guide for the Care and Use of Laboratory Animals.

### Isolation and culture of primary BMMs

Female C57BL/6 mice aged 6 weeks were sacrificed, and sterilized instruments were used to isolate the femur and tibia of the mice and peel off the attached tissue. After the ends of the femur and tibia were cut, the bone marrow was flushed out into α-MEM containing 30 ng/mL M-CSF. The suspended primary cells were repeatedly pipetted to mix evenly, and then cultured in an incubator (5% CO _2_, 37°C). The medium was changed every 48 h until the cells reached 80%–90% confluence. After wash with PBS buffer, the cells were digested with trypsin for several minutes. α-MEM was added, and then the cell suspension was centrifuged at 800 *g* for 5 min at room temperature. BMMs were then plated in 96-well plates at a density of 8×10 ^3^ cells per well. After 24 h of culture in α-MEM containing M-CSF (30 ng/mL) in the cell incubator, the medium was changed to osteoclast differentiation medium (unless otherwise specified, ‘osteoclast differentiation medium’ below refers to α-MEM containing 50 ng/mL RANKL and 30 ng/mL M-CSF). Cells were treated with different doses of MDHB (0, 5, 10, 20 μM) at the same time. The 0 μM MDHB group was set as the control group. When mature osteoclasts were observed in the control group, the cells were fixed with 4% paraformaldehyde and then stained with TRAP. Cells with more than 3 nuclei that are positive for TRAP staining are considered osteoclasts.

### Osteoblastogenesis assay *in vitro*

Mouse MC3T3-E1 MSCs were cultured in α-MEM supplemented with 10% FBS. Cells were then cultured in osteogenic medium (1 mM β-glycerophosphate, and 5 mM L-ascorbic acid 2-phosphate) with different concentration of MDHB (0, 10, 20 μM) for 7 days. The medium was changed every other day. Then cells were stained with the BCIP/NBT kit (CWBIO, Beijing, China) to detect ALP staining (Sigma-Aldrich).

### Bone resorption and F-actin ring formation of osteoclasts

After sterilization, bovine bone slices were placed in a 96-well plate. BMMs were seeded on bovine bone slices or in observation wells without bone slices at a density of 8×10 ^3^ cells per well. The cells were cultured with osteoclast differentiation medium until mature osteoclasts formed in the observed well. Then, MDHB at different doses was added to treat osteoclasts on bovine bone slices for 3 days. After wash with PBS, the bovine bone slices were removed and observed using a scanning electron microscope (Thermo Fisher Scientific, Waltham, USA). Quantitative analysis of the resorption area of bovine bone slices was performed using ImageJ software (NIH, Bethesda, USA).

BMMs were treated as described above. After that, the cells were fixed with 4% paraformaldehyde and permeabilized with 0.2% Triton X-100 for 5 min. Then, F-actin was stained with CoraLite 594 phalloidin (1:1000 in PBS; Proteintech, Chicago, USA). The nuclei of cells were stained with DAPI (1:1000 in PBS; Millipore Sigma, St Louis, USA). Finally, F-actin formation was observed under a fluorescence microscope (FV1200; Olympus, Tokyo, Japan) and quantitatively analyzed by ImageJ software.

### Cytotoxicity of MDHB on BMMs

BMMs were seeded in a 96-well plate at 2×10 ^4^ cells per well and cultured in α-MEM containing M-CSF (30 ng/mL) for 24 h. Then, different doses of MDHB were added to continue to treat the cells for 48 and 96 h. After that, a CCK-8 kit (YEASEN, Shanghai, China) was used to assay the cytotoxicity of MDHB on cells according to the manufacturer’s instructions A microplate reader (BioTek Instruments, Winooski, USA) was used to detect the optical density (OD) value of the cells at a wavelength of 450 nm.

### Immunofluorescence microscopy

After being seeded over the cell slides and cultured in the cell incubator for 24 h, cells were pretreated with different concentrations of MDHB for 2 h and were then stimulated with 50 ng/mL RANKL for 30 min. Subsequently, the cells were fixed with 4% paraformaldehyde and permeabilized with 0.2% Triton X-100 for 5 min. After wash with PBS, the anti-p65 primary antibody (1:200; Abcam) was added and incubated with fixed cells for 12 h at 4°C in the dark. Cells were washed with PBS three times and then incubated with horse radish peroxidase (HRP)-conjugated secondary antibody (1:5000; Abcam) at room temperature for 1 h in the dark. Next, DAPI was added to stain the nuclei. Finally, the nuclear translocation of p65 was determined by the fluorescence microscopy.

### Real-time RT-PCR analysis

After being seeded into 12-well plates, BMMs were treated with or without MDHB (20 μM) for 5 days in osteoclast differentiation medium. Then, 1 mL of TRIzol Reagent (Invitrogen, Carlsbad, USA) was added to each well, and the cells were lysed by repeated pipetting. Then, total RNA extraction was performed with an ultrapure RNA kit (CWbio, Beijing, China).

The extracted total cellular RNA was used for subsequent reverse transcription using the High-Capacity cDNA Reverse Transcription Kit from Applied Biosystems (Foster City, USA). RT-PCR was performed using a SYBR Premix Ex Tag kit (TaKaRa Biotechnology, Dalian, China) to further quantify the expression of genes. The expression levels of all genes were normalized to the expression of *Gapdh*. Sequences of the primers used for PCR are listed in Table 1.

### Western blot analysis

BMMs were seeded into 6-well plates for different treatments. Following washing the cells with PBS, RIPA lysis buffer (FdBio, Hangzhou, China) mixed with protease inhibitors (1:100; FdBio) was added to lyse the cells at 4°C for 60 min. The cell lysate was collected and centrifuged at 16,000 *g* at 4 °C for 15 min. The supernatant was collected as protein sample. BCA kit (Beyotime) was used to determine the protein concentration. Then, 4× SDS-PAGE loading buffer was added to each sample, and the protein was denatured at 100°C. The sample was used for 10% SDS-PAGE (Bio-Rad, Hercules, USA) and then electrotransferred to a PVDF membrane. The PVDF membrane was blocked in TBST (50 mM Tris, pH 7.6, 150 mM NaCl, 0.1% Tween 20) containing 5% skimmed milk for 1 h at room temperature. Subsequently, the diluted primary antibody was incubated with the blocked PVDF membrane at 4°C for 12 h. Then, the membrane was washed 3 times with TBST for 10–15 min each time. Next, the secondary antibody corresponding to the species of primary antibody was diluted and incubated with the membrane for 60 min at 25°C. After three times of wash with TBST, the membranes were exposed using ECL developer solution under a Bio-Rad Imaging System (Bio-Rad). The captured images were analyzed using ImageJ software.

### Coimmunoprecipitation

In immunoprecipitation experiments, cells were cultured with different doses of DMHB (0, 10, 20 μM) for 6 h under RANKL stimulation. After lysis, the cells were centrifuged at 16,000 *g* for 10 min at 4°C. Anti-Nrf2 antibody was added to the collected supernatant and incubated for 12 h at 4°C. The antibody-antigen complex was then formed. After that, the antibody-antigen complex was incubated with A/G agarose beads (Beyotime) at 25°C for 15–30 min. After wash with the indicated buffer, the magnetic beads and antibody-antigen complexes were eluted at 95°C for 5 min. The sample was subsequently used for western blot analysis using anti-ubiquitination antibody to detect the ubiquitination level of Nrf2.

### Analysis of ROS production

Intracellular ROS levels were measured using an ROS assay kit (Beyotime) according to the manufacturer’s protocol. Briefly, BMMs in different pretreatments were treated with 2 μM DCFHDA (Beyotime), a fluorescent probe, and incubated at room temperature for 20 min. Then, the cells were stimulated with 50 ng/mL RANKL for 10 min. After washing and centrifugation, fluorescence intensity was measured by flow cytometry (BD Biosciences, Franklin Lakes, USA) using excitation/emission wavelengths of 488/525 nm. The mean fluorescence intensity was quantitatively analyzed.

### Ovariectomy (OVX)-induced osteoporosis in mice

Twenty healthy 10-week-old female C57BL/6J mice were randomly divided into 4 groups: sham, OVX, low-dose (Low) and high-dose (High) groups. The OVX operation was performed in the mice of the OVX, Low and High groups, while mice in the sham group received only a sham operation. Then, the corresponding mice in the Low and High groups were intraperitoneally injected with 2.5 or 10 mg/kg MDHB every 3 days for 6 weeks. At the same time, mice in the sham and OVX groups were also injected intraperitoneally with PBS as a control. Subsequently, all mice were sacrificed. The tibiae of mice were fixed in 4% paraformaldehyde and subject to subsequent histological and micro-CT analyses.

### LPS-induced calvaria osteolysis in mice

Fifteen healthy 10-week-old female C57BL/6J mice were randomly divided into 3 groups: LPS group, low-dose (Low) group, and high-dose (High) group. LPS (12.5 mg/kg body weight) was injected into the middle surface of calvaria of all mice every 2 days for 8 days. On the next day after each LPS injection, low dose (2.5 mg/kg) or high dose (10 mg/kg) of MDHB was injected intraperitoneally, respectively, into the Low group and the High group. PBS was injected intraperitoneally into the LPS group as a control at the same time. Subsequently, all mice were sacrificed. The calvaria of mice were fixed in 4% paraformaldehyde and subject to subsequent histological and micro-CT analyses.

### Micro-CT analysis

After removal of the soft tissue, a high-resolution micro-CT (SkyScan 1072; Bruker microCT, Kontich, Belgium) was used to determine the microstructure of calvaria and the proximal tibia of mice. The resident reconstruction program (SkyScan, Aartselaar, Belgium) was used to analyze the structural parameters in the square region of interest set at 0.5 mm from the tibial growth plate, including bone volume per tissue volume (BV/TV), trabecular thickness (Tb.Th), number (Tb.N), and separation (Tb.Sp). The porosity of calvaria was also analyzed.

### Bone histomorphometry

After fixation, the tibias were decalcified in 10% EDTA for 3 weeks and embedded in paraffin. Tissue sections (approximately 4 μm thick) were stained with TRAP (Abcam) and hematoxylin and eosin (H&E) (Abcam). Images of stained sections were observed under the microscope. The bone surface covered by osteoclasts (Oc. S/BS) in each sample and eroded surface/bone surface (ES/BS) were analyzed.

### Statistical analysis

Data are presented as the mean±SD. Prism 8 (GraphPad, San Diego, USA) was used for data visualization analysis. Student’s *t*-test or one-way ANOVA followed by Tukey’s *post hoc* analysis, where appropriate, was performed to compare the statistical differences. *P*<0.05 was considered statistically significant.

## Results

### MDHB attenuates osteoclast differentiation *in vitro*

**Figure 1:**
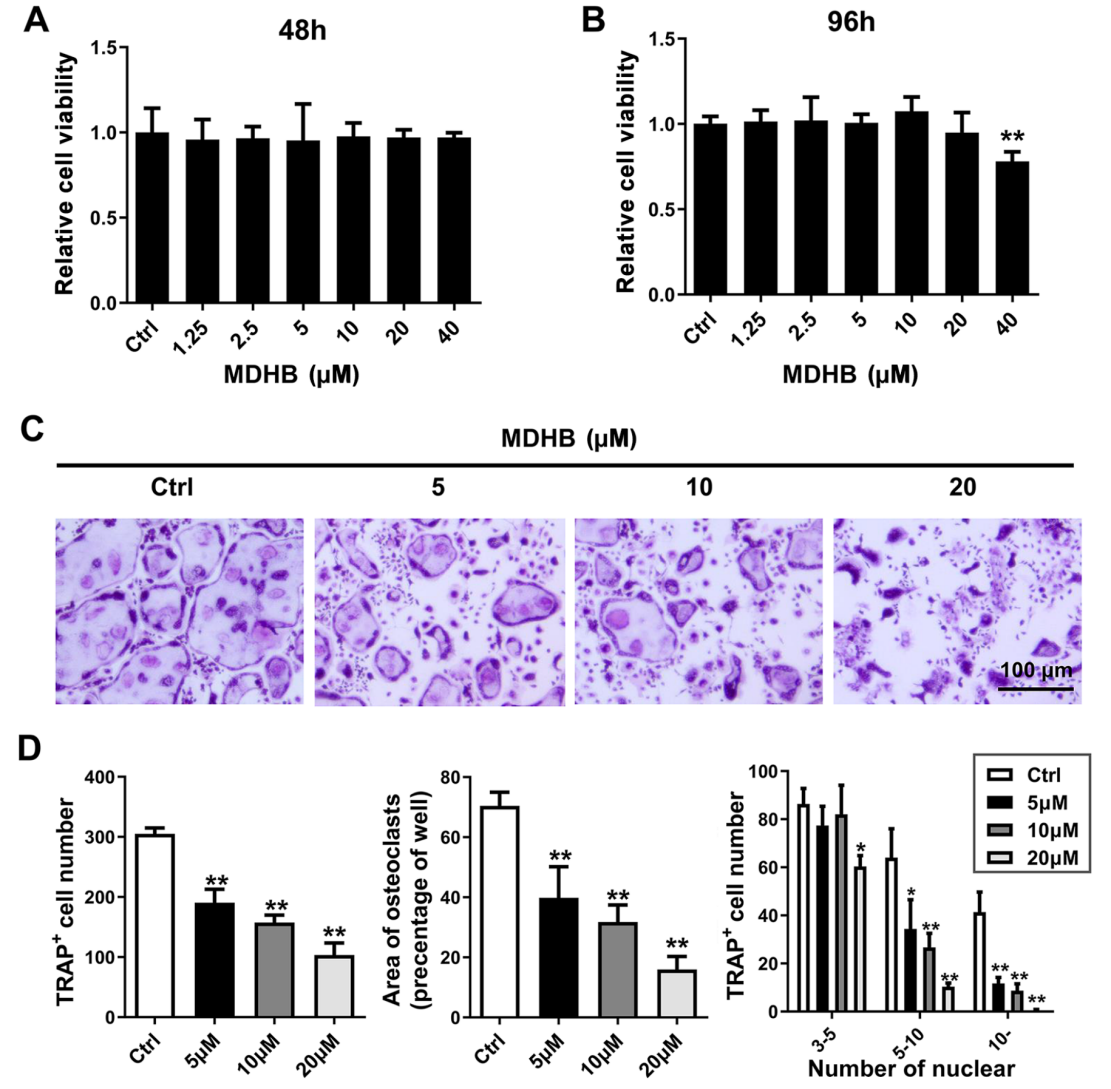
Figure 1 MDHB inhibited osteoclast differentiation *in vitro* (A,B) The effects of of MDHB on BMM proliferation. The effects of different doses of MDHB on BMM proliferation were assayed at 48 and 96 h. (C) TRAP staining of osteoclasts. BMMs differentiate into osteoclasts under stimulation of osteoclast differentiation medium with different doses of MDHB (0, 5, 10, and 20 μM). After multinucleated osteoclasts were observed in the 0 μM MDHB group, 4% paraformaldehyde was added to fix the cells. TRAP staining of osteoclasts was then performed. (D) Quantification of the number of osteoclasts, area of osteoclasts per well, and number of cells with different nuclei. DMSO (0.1%) was added to the 0 μM MDHB group as a control. Verifications for all data were repeated at least 3 times. Scale bar =100 μm. * P<0.05, ** P<0.01.

First, the effect of MDHB on BMM viability was tested by the CCK-8 assay. It was found that 20 μM MDHB did not affect BMM proliferation at 48 and 96 h. However, 40 μM MDHB slightly inhibited cell viability ( Figure 1A,B). To further determine the effects of MDHB on osteoclast differentiation, BMMs were cultured in osteoclast differentiation medium with different doses of MDHB (0, 5, 10, 20 μM) for 5 days. The number of TRAP-positive osteoclasts was decreased from 300±13.5 cells/well (with 0 μM MDHB) to 100±12.3 cells/well (with 20 μM MDHB) in a dose-dependent manner ( Figure 1C,D). The area of mature osteoclasts was also suppressed by MDHB treatment in a dose-dependent manner. Furthermore, MDHB exhibited significant inhibitory effects on the formation of osteoclasts with more than 10 nuclei. In addition, MDHB did not suppress the osteoblastogenesis according to the results of ALP staining ( Supplementary Figure S1A). Meanwhile, MDHB treatment showed no effect on the expression levels of osteoblast-related genes such as *OCN*, *ALP*, *COL1a* and *Runx2* ( Supplementary Figure S1B). Collectively, our results showed that MDHB inhibited RANKL-induced osteoclastogenesis.

### MDHB suppresses the bone resorption capacity of mature osteoclasts

**Figure 2:**
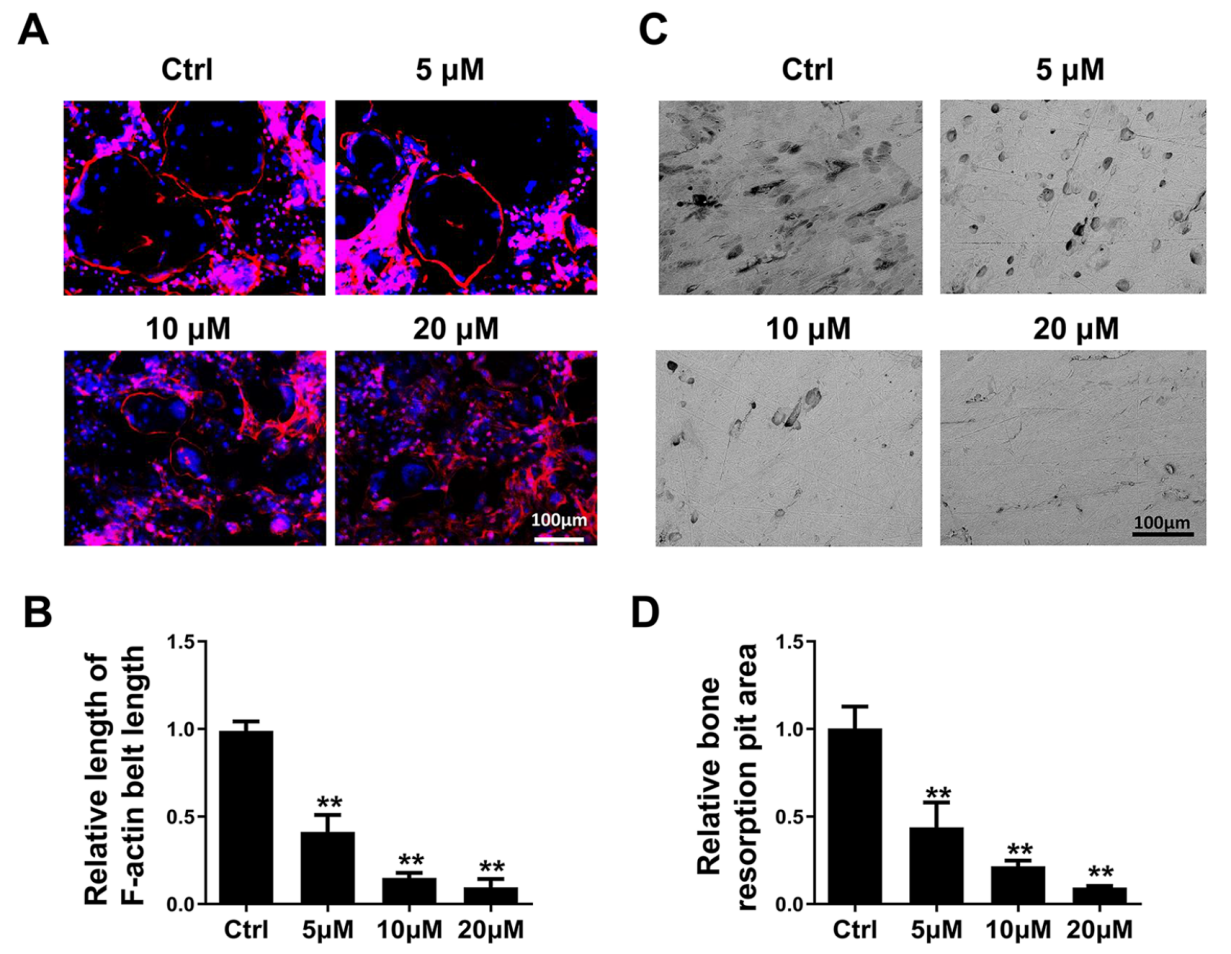
Figure 2 MDHB inhibited bone resorption of osteoclasts *in vitro* (A) Morphology observation of osteoclasts. BMMs were cultured in osteoclast differentiation medium until multinucleated osteoclasts appeared and then treated with different doses of MDHB for 72 h. After fixation, phalloidin was used for F-actin staining of cells, and then the cells were examined under a fluorescence microscope. (B) Quantitative analysis of the length of the F-actin ring of osteoclasts. (C) BMMs were plated on bovine bone slices and processed as in A. Bovine bone slices were removed, and the bone resorption lacuna were observed with a scanning electron microscope. (D) Quantitative analysis of bone resorption area. Verifications for all data were repeated at least three times. DMSO (0.1%) was added to the 0 μM MDHB group as a control. Scale bar =100 μm. * P<0.05, ** P<0.01.

The functions of osteoclasts in attachment to the bone surface and bone resorption are dependent on the formation of F-actin. We thus explored whether MDHB treatment could attenuate the function of osteoclasts by damaging the F-action belt. Immunofluorescence staining of mature osteoclasts showed that the formation of osteoclastic F-actin was damaged by MDHB in a dose-dependent manner ( Figure 2A,B). In addition, the bone resorption ability of mature osteoclasts was also decreased under MDHB treatment ( Figure 2C,D). These data indicate that MDHB suppresses the bone resorption capacity of mature osteoclasts.

### MDHB inhibits the expressions of osteoclast-related genes

**Figure 3:**
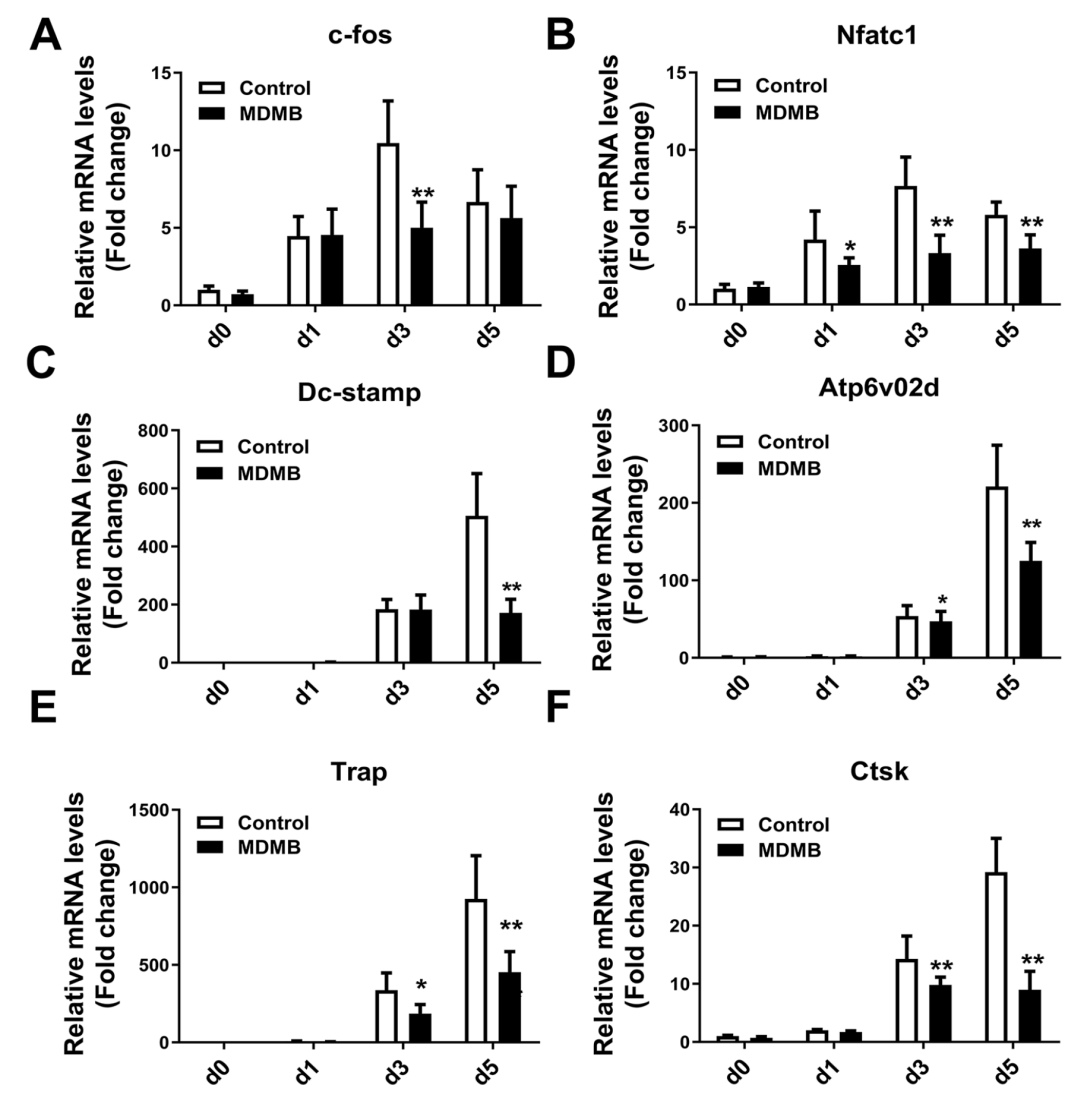
Figure 3 MDHB downregulated expression levels of osteoclast-related genes (A–F) The effect of MDHB on the expressions of osteoclast-related genes. BMMs were cultured in osteoclast differentiation medium with or without MDHB (20 μM). Total RNA was extracted on Days 0, 1, 3, and 5 of osteoclast differentiation, and RT-PCR was used to detect the expressions of osteoclast-related genes, including c-Fos, Nfatc1, Dc-stamp, Atp6v02d, Trap, and Ctsk. DMSO (0.1%) was added to the 0 μM MDHB group as a control. Verifications for all data were repeated at least 3 times. * P<0.05, ** P<0.01.

Osteoclast-related genes, such as *c-Fos*, *Nfatc1*, *Dc-stamp*, *Atp6v02d*, *Trap*, and *Ctsk*, in BMMs are upregulated under RANKL stimulation, and these genes encode proteins with vital functions during RANKL-induced osteoclastogenesis. We extracted total RNA from BMMs on the 1 ^st^, 2 ^nd^ and 3 ^rd^ days of culture in osteoclast differentiation medium with or without MDHB (20 μM). RT-PCR analysis showed that RANKL stimulation upregulated the mRNA levels of osteoclast-related genes ( Figure 3A–F). However, MDHB treatment suppressed the mRNA levels of genes upregulated by RANKL ( Figure 3A–F). These data indicate that MDHB inhibits the mRNA expressions of osteoclast-related genes.

**Figure 4:**
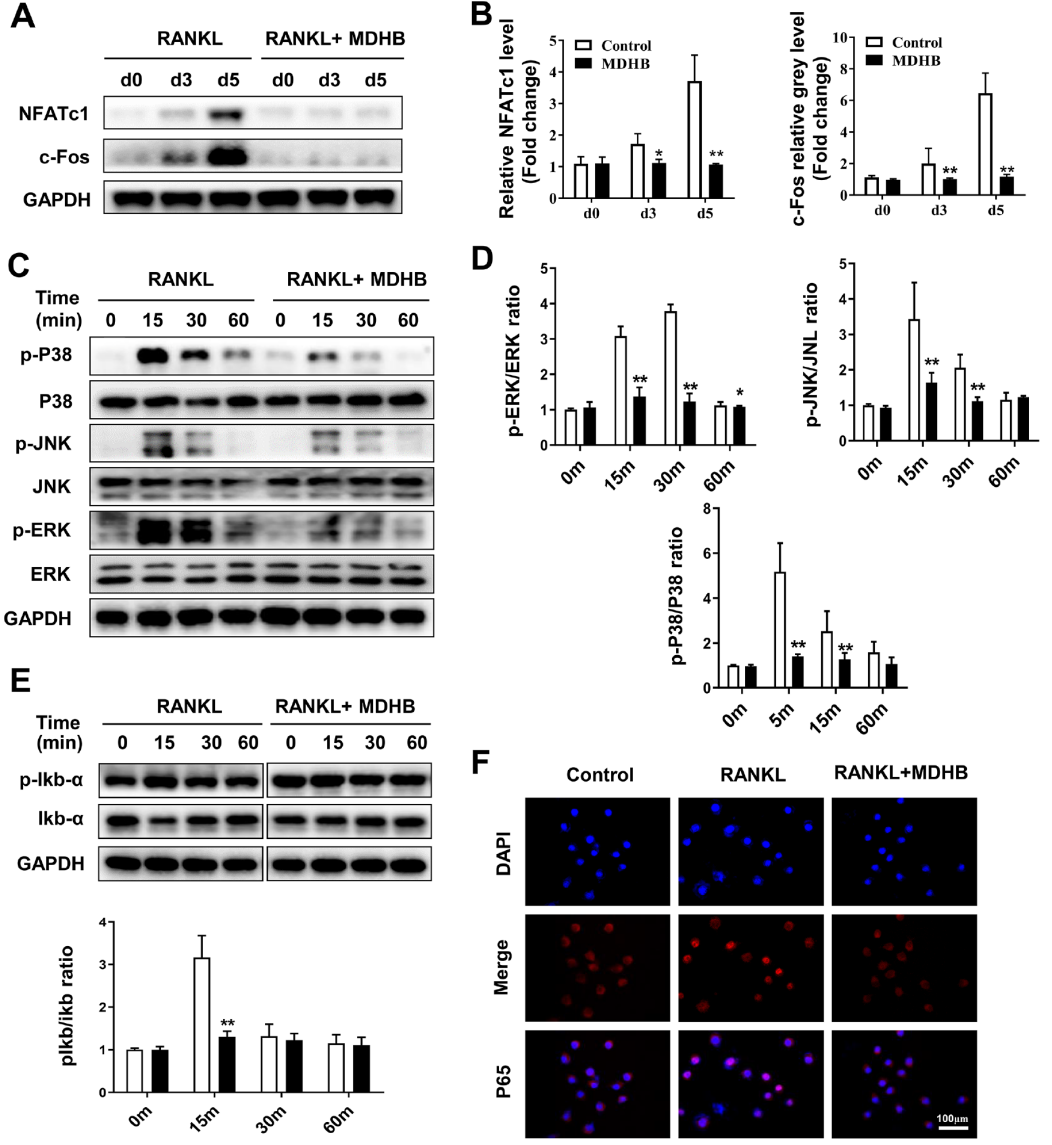
Figure 4 MDHB suppressed the activation of MAPK and NF-κB pathways *in vitro* (A) The effect of MDHB on the expressions of osteoclast-related proteins. BMMs were seeded on a 6-well plate and then cultured in osteoclast differentiation medium with or without 20 μM MDHB. Total protein was extracted on the 0, 3 rd and 5 th days of osteoclast differentiation. Western blot analysis was used to detect the expressions of osteoclast-related proteins, including NFATc1 and c-Fos. (B) Quantitative analysis of the gray levels of proteins using ImageJ software. (C,E) The effect of MDHB on the protein phosphorylation levels of the MAPK and NF-κB pathways. BMMs were pretreated with MDHB (0, 20 μM) for 2 h and then stimulated with RANKL (50 ng/mL) for 0, 15, 30, and 60 min. The protein phosphorylation levels of the MAPK and NF-κB pathways were detected by western blot analysis. (D) The gray levels of phosphorylated ERK, p38, JNK and IκBα-related total ERK, p38, JNK and IκBα were quantified using ImageJ software. (F) p65 nuclear translocation changes induced by MDHB. BMMs were pretreated with MDHB (0, 20 μM) for 2 h and then stimulated with RANKL (50 ng/mL) for 45 min. Immunofluorescence microscopy was performed to analyze p65 nuclear translocation according to the description in the Methods. DMSO (0.1%) was added to the 0 μM MDHB group as a control. Verifications for all data were repeated at least 3 times. Scale bar =100 μm. * P<0.05, ** P<0.01.

To determine the inhibitory effects of MDHB on MAPK pathway, we next explored whether MDHB affects the protein expression of NFATc1, c-Fos, CTSK and TRAP. After stimulation with RANKL and M-CSF for 0, 3, and 5 days, the protein expression levels of NFATc1, c-Fos, CTSK and TRAP were upregulated. However, MDHB treatment inhibited the upregulation by these four proteins ( Figure 4A,B and Supplementary Figure S2A,B). These data indicate that MDHB inhibits the protein expression of osteoclast-related genes.

### MDHB attenuates RANKL-induced activation of MAPK and NF-κB pathways

To further explore the mechanism of MDHB on osteoclast differentiation, we investigated whether it affects the MAPK and NF-κB pathways. As revealed by western blot analysis, the phosphorylation of p38, ERK, and JNK, relative to the total p38, ERK, and JNK, was upregulated by RANKL stimulation and significantly inhibited by MDHB ( Figure 4C,D). In the NF-κB pathway, RANKL-induced phosphorylation of IκB-α was inhibited by MDHB. Consistent with the inhibition of IκB-α, MDHB promoted the degradation of IκB-α ( Figure 4E). In addition, p65 nuclear translocation stimulated by RANKL was suppressed by MDHB ( Figure 4F). Furthermore, the phosphorylation of p65 was also inhibited by MDHB (Supplementary Figure S2C,D). Taken together, MDHB suppressed both MAPK and NF-κB pathway activation.

### MDHB reduces ROS levels by promoting Nrf2-induced antioxidative activity

**Figure 5:**
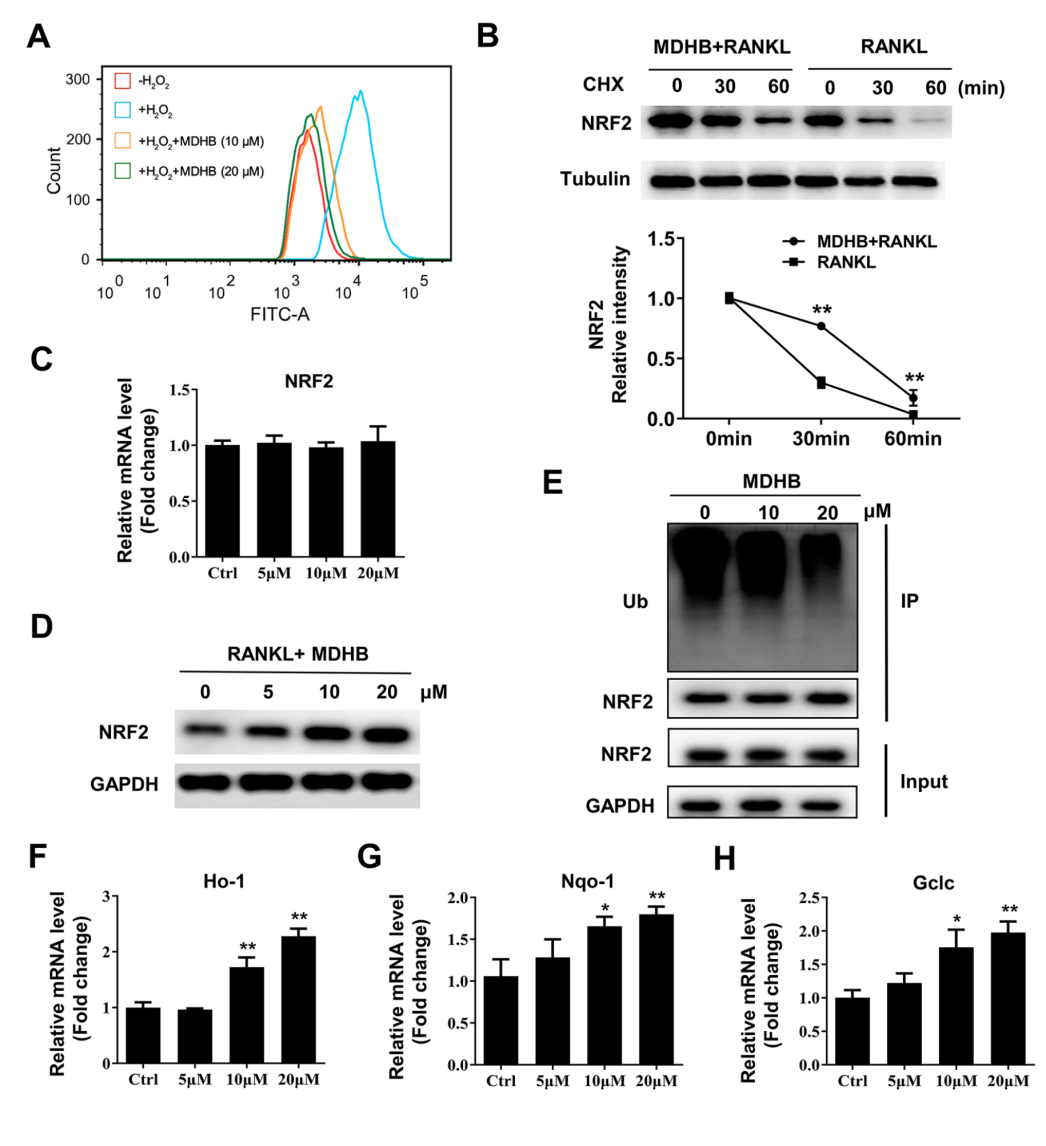
Figure 5 MDHB promoted ubiquitination-mediated Nrf2 degradation (A) Intracellular ROS analysis. BMMs were pretreated with different doses of MDHB (0, 10, 20 μM) for 2 h and then stimulated with H 2O 2 for 10 min. The level of intracellular ROS was analyzed by flow cytometry. (B) The effect of CHX on protein expression of NRF2. BMMs were pretreated with MDHB (0 or 20 μM) in osteoclast differentiation medium for 12 h and then treated with CHX (50 ng/mL) for 0, 30, or 60 min. Western blot analysis was performed to analyze the protein expression of NRF2. (C) Gene expression of NRF2. BMMs were treated with different doses of MDHB (0, 5, 10, and 20 μM) in osteoclast differentiation medium for 3 days. The gene expression of NRF2 was detected by RT-PCR. (D) The effect of MDHB on protein expression of NRF2. BMMs were treated with different doses of MDHB (0, 5, 10, and 20 μM) in osteoclast differentiation medium for 3 days. The protein expression of NRF2 was detected by western blot analysis. (E) The effect of MDHB and M-CSF on ubiquitination (Ub) of Nrf2. BMMs were pretreated with MDHB (0, 10, and 20 μM) and M-CSF (30 ng/mL) for 6 h before RANKL (50 ng/mL) was added. The ubiquitination of Nrf2 was determined by immunoprecipitation using anti-Nrf2 antibodies. (F-H) The effect of MDHB on gene expression levels of Ho-1, Nqo-1 and Gclc. BMMs were treated with different doses of MDHB (0, 5, 10, and 20 μM) in osteoclast differentiation medium for 3 days. The gene expression levels of Ho-1, Nqo-1 and Gclc were detected by RT-PCR. DMSO (0.1%) was added to the 0 μM MDHB group as a control. Verifications for all data were repeated at least 3 times. * P<0.05, ** P<0.01.

Because of the antioxidative property of MDHB, we next determined whether it affects ROS levels. BMMs were treated with H _2_O _2_ for 10 min in the presence of different doses of MDHB (0, 10, and 20 μM). Flow cytometric analysis of BMMs revealed that the increase in cellular ROS levels induced by H _2_O _2_ was significantly downregulated by MDHB treatment in a dose-dependent manner ( Figure 5A). Nrf2 is a key transcription factor that promotes the gene expression of antioxidant enzymes such as HO-1 and GSR to reduce ROS levels [28]. Furthermore, MDHB significantly attenuated Nrf2 protein degradation in the presence of the protein synthesis inhibitor cycloheximide ( Figure 5B). However, the mRNA expression of *Nrf2* was not affected by MDHB ( Figure 5C). Meanwhile, we found that MDHB treatment upregulated the protein expression of NRF2 in a dose-dependent manner ( Figure 5D). Immunoprecipitation results showed that MDHB treatment decreased the ubiquitination of Nrf2, which indicated that MDHB promoted Nrf2 protein expression by downregulating ubiquitination-mediated proteasomal degradation ( Figure 5E). In addition, the mRNA and protein expression levels of Ho-1, Gclc, and Nqo-1 were also upregulated by MDHB ( Figure 5F–H and Supplementary Figure S2E,F). Collectively, these results indicate that MDHB reduces ROS levels in BMMs by upregulating the expression of Nrf2.

### MDHB attenuates LPS-induced calvarial bone loss *in vivo*

**Figure 6:**
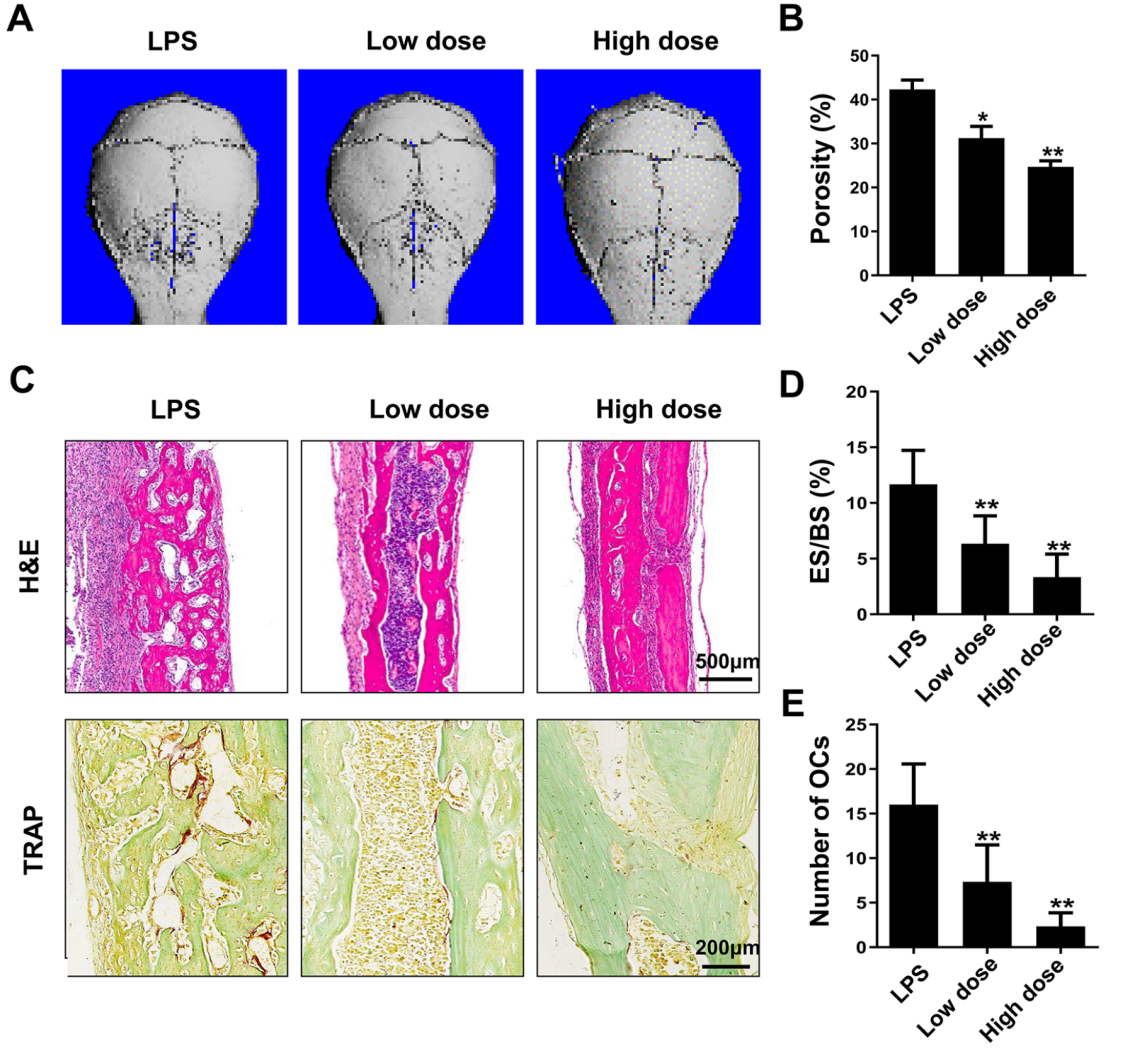
Figure 6 MDHB inhibited LPS-induced calvarial bone loss (A) Micro-CT analysis of mouse calvarial specimens in the LPS, low-dose and high-dose groups. (B) Quantitative analysis of the porosity of the calvaria of the 3 groups of mice based on micro-CT. (C) HE and TRAP staining of calvarial slices. After decalcification, the calvarial slices of the three groups of mice were stained with HE and TRAP. (D) Quantitative analysis of the number of osteoclasts per field of tissue and eroded surface (ES/BS) in sections stained by TRAP. * P<0.05, ** P<0.01.

Next, we investigated whether MDHB treatment affects osteoclast-induced bone loss. A mouse model of LPS-induced inflammatory bone loss due to elevated osteoclast activity was constructed. Micro-CT analysis confirmed that the porosity was lower in the low-dose and high-dose MDHB treatment groups than in the LPS group, indicating that MDHB successfully attenuated LPS-induced bone loss ( Figure 6A,B). Furthermore, H&E staining and TRAP staining showed that MDHB reduced LPS-induced bone erosion and increased the number of osteoclasts ( Figure 6C–E). These data indicate that MDHB attenuates LPS-induced calvarial bone loss *in vivo*.

### MDHB suppresses OVX-induced osteoporosis *in vivo*

**Figure 7:**
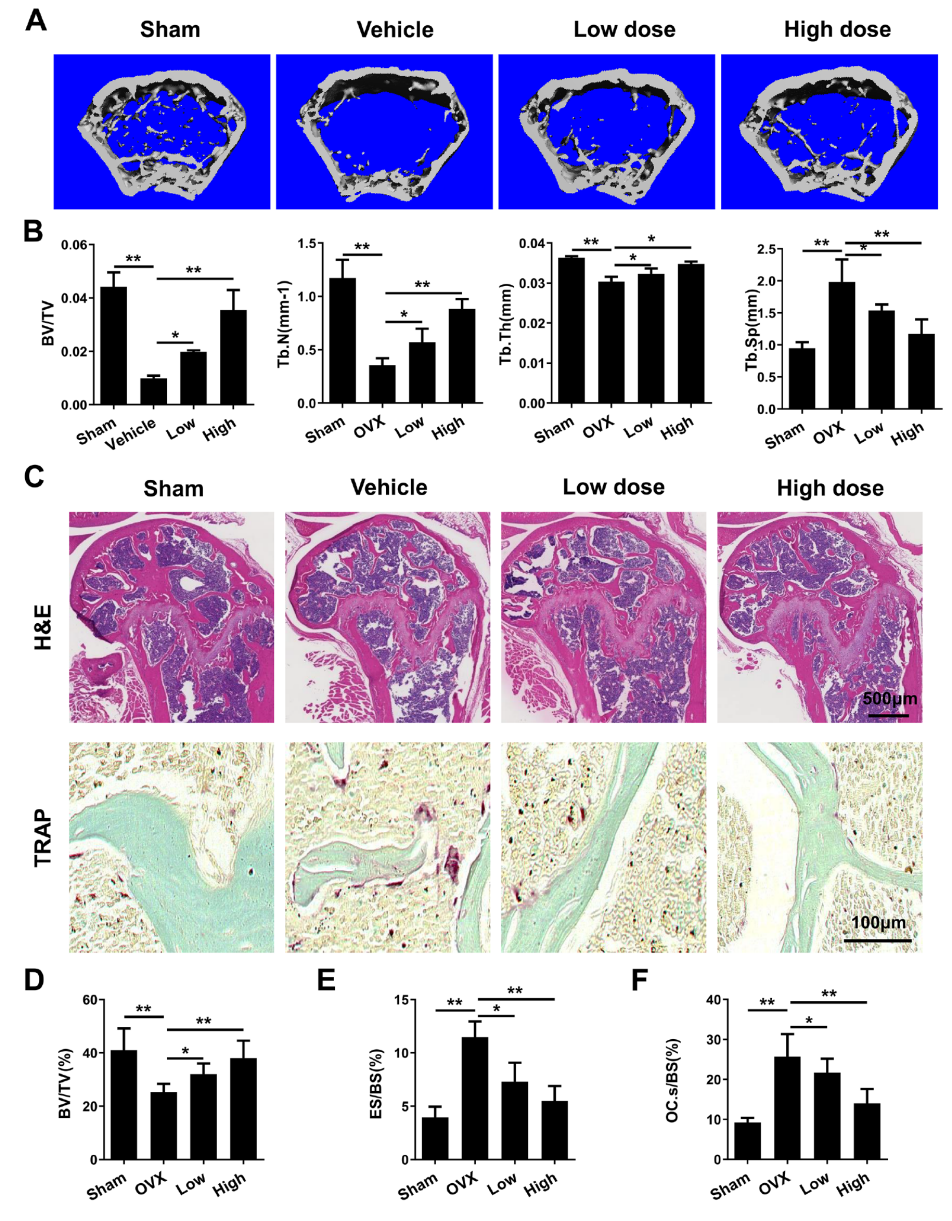
Figure 7 MDHB inhibited OVX-induced osteoporosis (A) Micro-CT analysis of mouse tibias specimens in the sham, vehicle, low-dose, and high-dose groups. (B) Quantitative analysis of bone volume/tissue volume (BV/TV), trabecular number (Tb.N), trabecular thickness (Tb.Th), and trabecular separation (Tb.Sp) of the 4 groups of mouse femurs based on micro-CT. (C) HE and TRAP staining of tibias. After decalcification, the tibias of the 4 groups of mice were stained with HE and TRAP. (D) Quantitative analysis of bone volume/tissue volume (BV/TV), eroded surface (ES/BS), and the number of osteoclasts per field of tissue (Oc. S/BS) based on TRAP staining. * P<0.05, ** P<0.01.

In addition, the potential treatment effect of MDHB in an OVX-induced osteoporosis mouse model was further explored. Four weeks after OVX or sham operation, mice were intraperitoneally injected with MDHB or vehicle every 3 days. Micro-CT analysis of mouse tibias showed significant decreases in BV/TV, Tb.N, and Tb.Th in the OVX group compared with those in the sham group, which were inhibited by MDHB at both low and high doses ( Figure 7A,B). Meanwhile, the increase in Tb.Sp in the OVX group was also inhibited by MDHB treatment compared with that in the sham group ( Figure 7A,B). Our results indicate that MDHB has a therapeutic effect on OVX-induced osteoporosis. Furthermore, H&E staining showed that MDHB treatment prevented the reduction of bone loss in OVX mice ( Figure 7C,D). TRAP staining analysis also indicated that MDHB treatment inhibited the increase in the number of osteoclasts induced by OVX ( Figure 7C–F). These data indicate that MDHB suppresses OVX-induced osteoporosis *in vivo* .

## Discussion

Osteoporosis is a well-known disease characterized by bone loss, which is common in postmenopausal women and elderly men [29]. Excessive osteoclastic bone resorption and suppressed osteogenic bone formation promote an imbalance in bone remodeling, further causing osteoporosis [30]. Some anti-osteoporosis drugs have been developed, including anti-osteoclast drugs, such as bisphosphonates, estrogen, and RANKL inhibitors [31]. However, these drugs have certain side effects. For example, bisphosphonates can be accompanied by adverse reactions in the digestive tract, and estrogen might induce cancer [32]. Therefore, it is necessary to develop safer and more effective osteoporosis drugs. In our study, we found that the anti-oxidant small molecule MDHB has the ability to inhibit osteoclast differentiation and bone resorption *in vitro* and suppresses OVX-induced osteoporosis in mice.

ROS have been reported to function as important regulators of osteoclast differentiation. After stimulation by RANKL, the TRAF6/Nox1 signaling pathway is activated, leading to increased ROS production in BMMs [12]. However, accumulated ROS, specifically H _2_O _2_, promote osteoclast differentiation by activating key pathways, such as the NF-κB and MAPK pathways [ 8, 33] . Numerous studies focusing on the emerging role of the MAPK and NF-κB pathways have shown that ERK, JNK, p38, and p65 are key regulators of osteoclast differentiation and affect the function of important transcription factors, especially NFATc1 and c-Fos, further affecting the expressions of osteoclast-related genes [ 34– 36] . Additionally, decreased levels of ROS production mediated by the oxidant scavenger N-acetylcysteine or the antioxidant small molecule pseurotin A were found to inhibit MAPK pathway activation during RANKL-induced osteoclastogenesis [ 12, 33] . MAPK phosphatases (MKPs) function as MAPK inhibitors via dephosphorylation. A previous study indicated that ROS can oxidize tyrosine phosphatases, inducing the inhibition of MKPs and activating MAPKs [37]. Meanwhile, ROS promote the homodimerization of LC8, leading to the dissociation of LC8 and IκBα and subsequently increasing the phosphorylation and degradation of IκBα, thus stimulating the nuclear translocation of NF-κB dimers [ 38, 39] . Our results showed that MDHB suppressed the RANKL-mediated phosphorylation of ERK, JNK, and p38 and promoted the degradation of IκBα, which led to the inhibition of NFATc1 and c-Fos. Furthermore, MDHB could reduce ROS, indicating that it inhibits the MAPK and NF-κB pathways by reducing ROS production. MDHB further suppressed the expressions of NFATc1 and c-Fos.

Nrf2 functions as a redox-sensitive basic leucine zipper transcription factor by binding to AREs to positively regulate the expression of numerous antioxidant and phase II detoxifying enzymes, such as HO-1, GSH, NQO-1, Prdx1, and TrxR1 [ 18, 40– 42] . A previous study showed that Nrf2 deficiency in BMMs promotes osteoclast differentiation and bone resorption by increasing ROS accumulation [18]. In addition, the Nrf2 activators RTA-408 and schisandrin A have been shown to inhibit osteoclastogenesis [ 21, 43] . Overall, these studies suggest that Nrf2 is a key transcription factor during osteoclastogenesis and functions by promoting ROS scavenging. The ubiquitin-mediated proteasomal degradation of Nrf2, regulated by Keap1, is important for Nrf2 activity [ 44, 45] . Under normal conditions, Keap1 binds to Nrf2 and promotes the formation of the E3-ubiquitin ligase complex with Nrf2, leading to proteasomal degradation of Nrf2. Under oxidative stress, the cysteine residues of Keap1 are modified, resulting in reduced binding between Keap1 and Nrf2. Then, the degradation of Nrf2 is inhibited, and Nrf2 translocates to the nucleus to induce the expression of antioxidant genes. What’s more, a recent study also showed that Nrf2 was identified as the epigenetic regulator by modulating miRNA levels during inflammatory diseases such as LPS-induce bone loss. The Nrf2 activator effectively promotes the binding of Nrf2 and miR-214-3p, leading to downregulation of miR-214-3p and further inhibition of osteoclastogenesis [46]. In addition, another study also proposed that Nrf2 may be involved in the STING-TRAF6 axis to regulate osteoclast differentiation [21]. Meanwhile, a study also found the Nrf2 activity in osteocytes during the regulation of osteocyte gene expression and maintenance of bone homeostasis [47]. In our study, we found that MDHB inhibited the ubiquitination of Nrf2 to upregulate Nrf2 protein expression in BMMs upon RANKL stimulation in a dose-dependent manner. Consistently, the expression levels of the antioxidant genes *Ho-1*, *Gclc*, and *Nqo-1* were also upregulated by MDHB treatment.

Although we found that MDHB has a promotive effect on Nrf2 expression, the molecular mechanism underlying this effect is still unknown. Various molecules in addition to Keap1 have been reported to be involved in Nrf2 expression and activity. For example, the E3 ubiquitin ligase Hrd1 is responsible for the attenuation of Nrf2 by enhancing Nrf2 ubiquitination during liver cirrhosis [48]. A previous study also showed that octyl-itaconate activates Nrf2 signaling by suppressing Hrd1 during RANKL-induced osteoclastogenesis, suggesting that Hrd1 might be a key regulator of Nrf2 during osteoclastogenesis [49]. Another study further suggested that p65 promotes HDAC3 interaction with CBP and competitively inhibits the Nrf2-CBP binding-induced activation of ARE in acute inflammation. Moreover, p65 can bind with Keap1 to suppress Nrf2 transcription activities, upregulate ROS levels, and promote inflammation [50]. In our study, we showed that MDHB effectively inhibited the NF-κB pathway and upregulated Nrf2 expression, which indicated that it might promote Nrf2 through the NF-κB pathway. In addition, inhibiting Nrf2 transcription induced ROS accumulation and promoted the activation of NF-κB. Given the important roles of Nrf2 and NF-κB in osteoclastogenesis, the positive feedback between Nrf2 and NF-κB could be a key biochemical process in osteoclasts. It is worth exploring whether MDHB attenuates RANKL-induced osteoclastogenesis by blocking the positive feedback loop between Nrf2 and NF-κB.

In summary, our study demonstrates for the first time that MDHB promotes the expression of Nrf2 by reducing ubiquitination-induced proteasomal degradation of Nrf2 and reducing ROS levels, leading to inhibition of the activation of the MAPK and NF-κB pathways. These processes eventually result in the suppression of RANKL-induced osteoclastogenesis. Moreover, MDHB has a therapeutic effect on LPS-induced calvarial bone loss and OVX-induced osteoporosis in mice. Due to its low cytotoxicity, MDHB might be a safe and effective drug for osteoporosis treatment. In addition, developing a drug to block Nrf2 proteasomal degradation and promote Nrf2 expression could be useful for osteoporosis treatment in the future.

## Supplementary Data

Supplementary Data is available at *Acta Biochimica et Biphysica Sinica* online.
